# Automating the application of smart materials for protein crystallization

**DOI:** 10.1107/S1399004714027643

**Published:** 2015-02-26

**Authors:** Sahir Khurshid, Lata Govada, Hazim F. EL-Sharif, Subrayal M. Reddy, Naomi E. Chayen

**Affiliations:** a Computational and Systems Medicine, Department of Surgery and Cancer, Faculty of Medicine, Imperial College London, London SW7 2AZ, England; b Department of Chemistry, Faculty of Engineering and Physical Sciences, University of Surrey, Guildford, Surrey GU2 7XH, England

**Keywords:** protein crystallization, molecularly imprinted polymers, smart materials

## Abstract

The fabrication and validation of the first semi-liquid nonprotein nucleating agent to be administered automatically to crystallization trials is reported. This research builds upon prior demonstration of the suitability of molecularly imprinted polymers (MIPs; known as ‘smart materials’) for inducing protein crystal growth. Modified MIPs of altered texture suitable for high-throughput trials are demonstrated to improve crystal quality and to increase the probability of success when screening for suitable crystallization conditions. The application of these materials is simple, time-efficient and will provide a potent tool for structural biologists embarking on crystallization trials.

## Abstract 2

The first semi-liquid, non-protein nucleating agent for automated protein crystallization trials is described. This ‘smart material’ is demonstrated to induce crystal growth and will provide a simple, cost-effective tool for scientists in academia and industry.

## 1. Introduction   

Statistics indicate that the success rate of obtaining diffraction-quality crystals from purified proteins has stagnated at ∼20% for over a decade (Target Track PSI; http://sbkb.org/tt/). This has fuelled methods development and advances including the application of porous nucleants (Chayen *et al.*, 2006 ▶; Khurshid *et al.*, 2014 ▶), the lipidic cubic phase (Nollert, 2004 ▶; Caffrey & Cherezov, 2009 ▶), novel microfluidic platforms (Zheng *et al.*, 2003 ▶; Shim *et al.*, 2007 ▶), commercial screens of greater potency (Newman *et al.*, 2005 ▶; Gorrec, 2009 ▶) and novel seeding protocols (Georgiev *et al.*, 2006 ▶; D’Arcy *et al.*, 2007 ▶), which have been reported with success. In order for methodological advances to be of benefit to the wider community it is imperative that they be adapted to high throughput.

This article details the fabrication and validation of the first semi-liquid nonprotein nucleant compatible with automated crystallization trials. Nucleants are materials which induce heterogeneous nucleation of protein crystals in a controlled manner. Nucleant research has advanced significantly since pioneering studies in 1988 (McPherson & Shlichta, 1988 ▶). The current trend for nonprotein porous materials has resulted in a plethora of candidate nucleants (Asanithi *et al.*, 2009 ▶; Rong *et al.*, 2004 ▶; Curcio *et al.*, 2003 ▶; Sugahara *et al.*, 2008 ▶; Kertis *et al.*, 2012 ▶; Saridakis & Chayen, 2009 ▶) and a commercial product. Several of these materials have been successful but are not readily amenable to automation.

**Figure 1:**
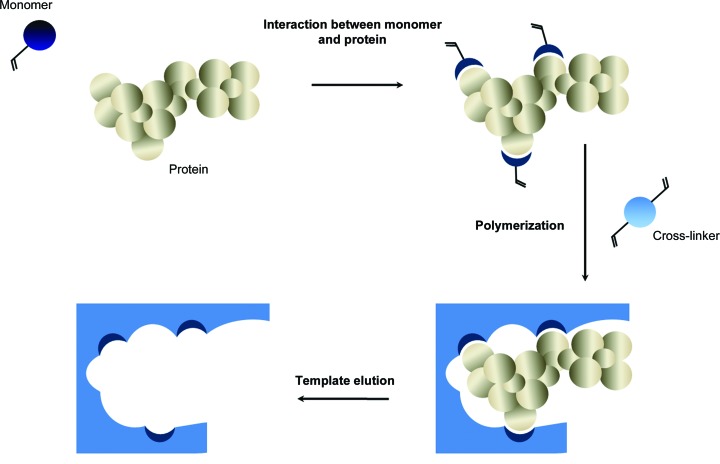
Fabrication of MIPs. Schematic illustration of the steps involved in preparing MIPs for crystallization studies. The initial assembly between the protein template and monomer is advanced through the presence of a polymerizing cross-linker. The protein is then eluted, leaving a protein-specific cavity known as a ‘ghost site’ (adapted from http://www.biotage.com/product-page/mips---molecularly-imprinted-polymers).

In 2011, we reported the application of molecularly imprinted polymers (MIPs; known as ‘smart materials’) as nucleants for protein crystallization (Saridakis *et al.*, 2011 ▶). These polyacrylamide-based nucleating agents were demonstrated to induce the nucleation of nine proteins under metastable conditions as well as increasing the number of crystal leads obtained from screening. MIPs were created by imprinting a protein in a polymer and then removing it, leaving behind specific cavities which would then rebind this protein when introduced into a crystallization trial (Fig. 1 ▶). It was initially envisaged that these materials would be specific, in that an MIP would only be suitable for the cognate protein it was imprinted with. In practice this has not been the case, with proteins of similar molecular weight to the cognate nucleating successfully. This correlation between the cavity/pore size and the hydrodynamic radius of protein molecules in solution is a recurring theme in heterogeneous nucleant research (Page & Sear, 2006 ▶).

The gel-like consistency of MIPs, similar to that of a high-molecular-weight PEG (and their efficacy as a nucleating agent) makes them promising candidates for automated crystallization trials. This very consistency is also a cause for concern, with preliminary trials indicating that MIPs would block robotic dispensing tips despite viscosity adjustments. Accordingly, the primary aim of this research was the production of a less viscous MIP which could be dispensed automatically whilst ensuring the integrity of the MIP as a nucleating agent.

The benefits of having a ‘standalone’ nucleant which can be added indiscriminately to crystallization trials, independent of the protein (or the crystallization cocktail for that matter), are numerous. Previous attempts at robotically dispensing heterogeneous nucleants have involved solid materials being crushed and dispensed as a suspensions (Thakur *et al.*, 2007 ▶; D’Arcy *et al.*, 2004 ▶; Stewart *et al.*, 2011 ▶). This raises questions as to which solvent to use, the optimal dilution of the suspension, the volume to dispense, whether this will dilute/bias the crystallization droplets and whether there is a reproducible amount of nucleant per suspension volume. If the nucleant is added to the protein stock prior to dispensing, the topographical features of the nucleant become saturated with protein, potentially reducing their efficacy. The application of MIPs bypasses these concerns.

## 2. Materials and methods   

### 2.1. Protein information   

Six proteins were tested. Model proteins were utilized for automated screening trials with MIPs to benchmark the technique. These included lysozyme (hen egg white; Sigma, catalogue No. L7651), thaumatin (from *Thaumatococcus daniellii*; Sigma, catalogue No. T7638), trypsin (bovine pancreatic; Sigma, catalogue No. T4665) and haemoglobin (bovine; Sigma, catalogue No. H2500). The model proteins were prepared at the required concentrations in deionized water. Two target proteins, *Chlamydia trachomatis* plasmid-encoded immunodominant antigen (Pgp3; 7 mg ml^−1^ in 220 m*M* sodium chloride, 50 m*M* Tris–HCl pH 7.5 with 5 m*M* DTT) and human macrophage migration inhibitory factor (MIF; 11 mg ml^−1^ in 20 m*M* Tris pH 7.5, 20 m*M* sodium chloride), were utilized for the automated optimization trials with MIPs.

### 2.2. Fabrication of modified MIPs   

Modified, less viscous MIPs were prepared for five proteins: haemoglobin, trypsin, lysozyme, Pgp3 and MIF. These MIPs were synthesized using acrylamide monomers (AA; 71.1 g mol^−1^; Sigma–Aldrich UK) and *N*,*N*′-methylenebis­acrylamide (bis-AA) as a cross-linker (154.18 g mol^−1^; Sigma–Aldrich UK). Acrylamide-based polymers are nitrogen-containing members of the acrylate family and are suitable imprinting matrices for biological molecules as they are water-compatible, economical, easily produced and can be derivatized to introduce functional groups (namely hydroxyl, carboxylate and amino groups) to better engineer complementary interactions between the template molecule and the polymer (Liao *et al.*, 1996 ▶).

5.4 mg (0.76 *M*) AA and 0.6 mg (38.9 m*M*) bis-AA cross-linker were mixed with 0.6 mg ml^−1^ template protein (haemoglobin, 64.5 kDa, 9.3 µ*M*; trypsin, 23.8 kDa, 25.2 µ*M*; lysozyme, 14.3 kDa, 41.96 µ*M*; Pgp3, 28 kDa, 30.36 µ*M*; MIF, 12.3 kDa, 48.78 µ*M*) along with Milli-Q water, initiator [2 µl of a 10%(*w*/*v*) ammonium persulfate APS solution, 8.77 m*M*; Sigma–Aldrich UK] and catalyst [2 µl of a 5%(*v*/*v*) *N*,*N*,*N*,*N*-tetramethylethyldiamine (TEMED) solution, 8.61 m*M*; Sigma–Aldrich UK] to give final volumes of 100 µl. The solutions were then purged with nitrogen for 5 min and allowed to polymerize overnight at room temperature (∼22°C). A final gel density of 6%T [percentage (*w*/*v*) of AA plus bis-AA in the final monomer solution] and a final cross-linking density of 10%C (percentage by mass of bis-AA relative to the total mass of AA plus bis-AA) was obtained for AA/bis-AA (*w*/*v*)s. The molar ratios of AA monomer and bis-AA cross-linker to template protein were 81 720:1 and 4183:1 for haemoglobin, 30 159:1 and 1544:1 for trypsin, 18 112:1 and 927:1 for lysozyme, 25 033:1 and 1281:1 for Pgp3 and 15 580:1 and 797:1 for MIF.

For every modified MIP created, a non-imprinted control polymer (NIP) was prepared in an identical manner but in the absence of template protein.

After polymerization, the modified MIPs were conditioned (as detailed in Supporting Information §S1.1) before being diluted in Milli-Q water at ratios of 1:2, 1:3 and 1:5 [MIP:water (*w*:*v*)] and stored at 4°C for crystallization trials. The subsequent rebinding effect of the conditioned and equilibrated MIPs and NIPs were characterized as detailed in Supporting Information §S1.2.

### 2.3. Crystallization robotics   

Mosquito (TTP Labtech, UK) and Oryx (Douglas Instruments, UK) robots were utilized to test the high-throughput addition of MIPs to sitting-drop vapour-diffusion crystallization trials. 96-well MRC plates were employed as standard (Molecular Dimensions, UK).

### 2.4. Preliminary trials   

Thaumatin and trypsin were employed for initial trials which involved determining (i) whether the modified MIPs prompted the nucleation of crystals, (ii) which of the MIP dilutions was most suitable, (iii) the optimal volume of MIPs to add to the crystallization trials and (iv) whether the total drop volume had any bearing on the efficacy of the nucleant. A series of identical drops were prepared corresponding to a known metastable condition for 30 mg ml^−1^ thaumatin (0.24 *M* sodium potassium tartrate, 0.1 *M* bis-tris propane pH 6.8) and 50 mg ml^−1^ trypsin [13%(*w*/*v*) PEG 8000, 0.1 *M* Tris pH 7.5]. 190, 185, 180 and 175 nl crystallization droplets were dispensed for each protein, where the protein and crystallization condition were mixed in a 1:1 ratio. Cognate MIPs volumes of 10, 15, 20 and 25 nl were added to these respective droplets, ensuring a total trial volume of 200 nl. 1:2, 1:3 and 1:5 samples of each cognate MIP were tested such that eight repeats were dispensed for each combination. This protocol was repeated for 400 nl trials, where 10, 20, 30 and 40 nl volumes of MIPs were added to 390, 380, 370 and 360 nl crystallization droplets, respectively. Two control drops were dispensed for each condition: one where no MIP was inserted and a second where an NIP was inserted instead of an MIP.

### 2.5. Automated screening trials   

Four model proteins and three MIPs were utilized for the automated screening trials. Commercially available 96-condition screens (Crystal Screen HT, Hampton Research USA) were dispensed for 30 mg ml^−1^ lysozyme, 30 mg ml^−1^ thaumatin, 50 mg ml^−1^ trypsin and 60 mg ml^−1^ haemoglobin. Protein was mixed in a 1:1 ratio with each screen condition to form a 180 nl drop. 20 nl of a 1:2 MIP sample was added robotically to this 180 nl drop volume, ensuring a total trial volume of 200 nl. Trypsin-MIP, lysozyme-MIP and haemoglobin-MIP were tested for each protein. Comparison was made with control drops which contained no MIPs or contained NIPs instead of MIPs.

### 2.6. Automated optimization trials   

One model protein (trypsin) and two target proteins (Pgp3 and MIF) were used to validate the modified MIPs for automated optimization trials. These trials involved the exploitation of known ‘hit’ conditions for each protein and the metastable zone of the phase diagram. The ‘hit’ conditions and the robotic determination of metastability are detailed in Supporting Information §§S1.3 and S1.4, respectively.

7 mg ml^−1^ Pgp3 and 11 mg ml^−1^ MIF were dispensed against their corresponding ‘hit’ conditions, forming 180 nl crystallization drops. The ‘hit’ condition was dispensed neat and in 5% dilutions down to 45%. Eight repeats were dispensed at each dilution. 20 nl of a 1:2 cognate MIP was then added to form 200 nl trials.

As an alternative, a working phase diagram (Khurshid *et al.*, 2014 ▶) was prepared for 50 mg ml^−1^ trypsin by dispensing 90 nl of the protein into 90 nl of its ‘hit’ condition (where the primary precipitant PEG 8000 was decreased in 1% steps from 16 to 10%). Eight repeats were dispensed of each concentration and 20 nl trypsin-MIP was added to each repeat. The same grid was repeated utilizing haemoglobin-MIP to determine how effective a noncognate MIP with a larger cavity would be.

### 2.7. Diffraction analysis   

The diffraction resolution limits of the crystals obtained from the automated optimization trials were determined using an ‘in-house’ Rigaku MicroMax-007 HFM X-ray generator operating at 40 kV and 30 mA with VHF optics, a Saturn 944+ CCD detector and an Oxford Cryosystems 700 liquid-nitrogen cryostream. Comparison was made with crystals obtained in control drops at the lowest supersaturation at which crystallization would occur spontaneously. 12 Pgp3 crystals and eight MIF crystals were tested.

## 3. Results and discussion   

The addition of MIPs to screening and optimization trials using robots was successfully demonstrated for a range of model and target proteins. Before discussing the results obtained from the automated screening and optimization trials, it is important to shed light upon the results obtained from the preliminary trials, as they provided the basis for performing these later experiments in an optimal fashion.

### 3.1. Preliminary trials   

The first result to bear in mind is that the modified MIPs were much less viscous than the original gel-like MIPs, with a consistency similar to that of a low-molecular-weight PEG. The modified MIPs reproducibly facilitated crystal growth under metastable conditions for thaumatin and trypsin. Each of the MIP dilutions was successful in this regard. The 1:2 and 1:3 samples in particular provided the most reproducible results, whilst the 1:5 sample was not as efficient at lower supersaturation. This lack of efficiency indicates a probable loss of MIP cavity integrity upon excessive dilution. The 1:2 sample, although the most viscous of the modified MIPs, did not block the dispensing tips, and as such a 1:2 MIP sample was chosen for all subsequent trials.

The optimal volume of MIPs to incorporate into trials was found to be ∼10% of the total droplet volume. This tallies with the research performed with the original gel-like MIPs. Although the crystallization outcomes of the trials performed with varying MIP volumes greater than 10% were similar, it is preferable to prevent excessive dilution of the crystallization droplet with the liquid nucleant. Furthermore, dispensing less than 10% becomes a technical challenge when the total drop volume is 200 nl, as the accuracy plummets drastically. The irregular results obtained with 10 and 15 nl nucleant volumes corroborate this. When the total drop volume is 400 nl this is not an issue. However, most laboratories screen using 200 nl drops to minimize protein consumption, and as such an 180 nl crystallization droplet with an added 20 nl MIPs volume is recommended.

The effect of drop volume on the nucleating ability of the MIPs was found to be negligible. The results obtained with 200 and 400 nl trial volumes were identical and extrapolate directly to the results obtained at 600 nl using the original gel-like MIPs. As such, if the researcher wishes to utilize a greater drop volume to optimize crystal size, decreased MIP efficacy will not be an issue.

### 3.2. Automated screening trials   

**Figure 2:**
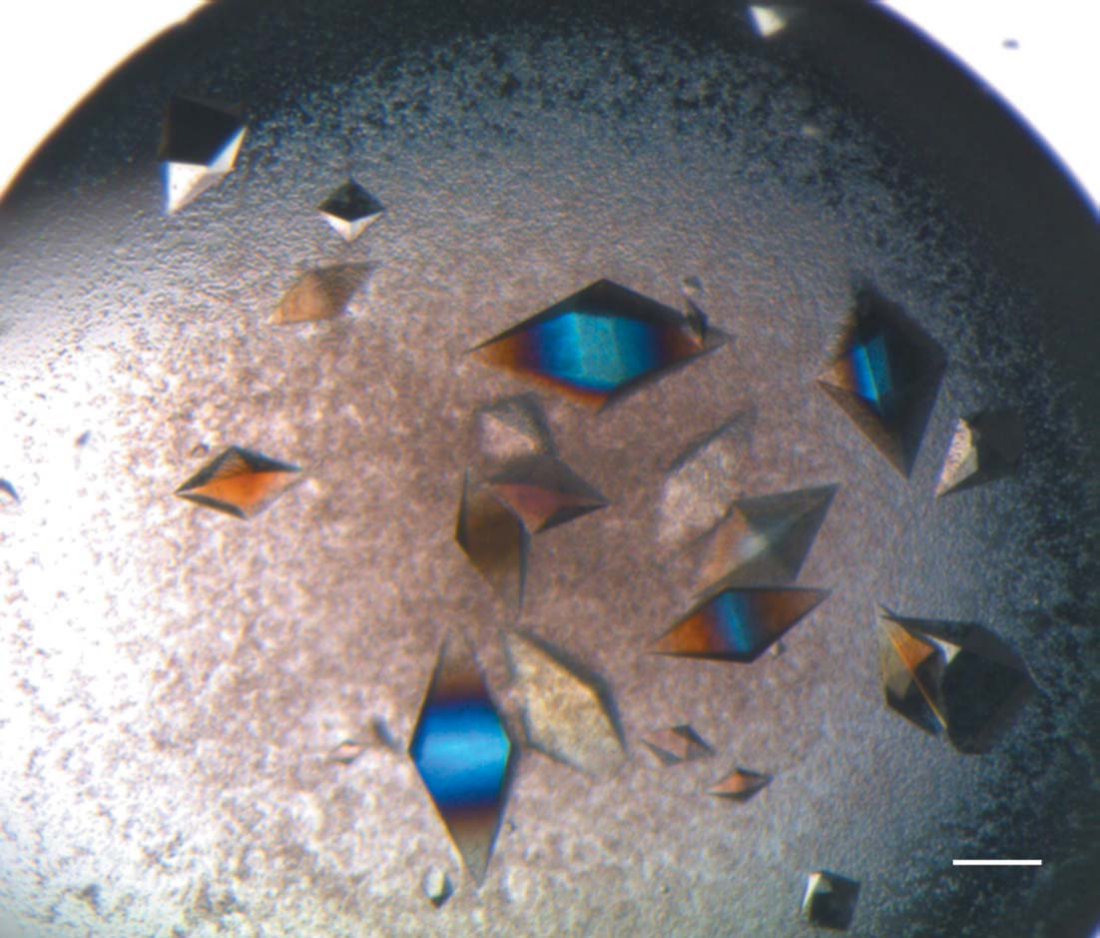
Thaumatin screening. Thaumatin crystals grown using a trypsin-MIP. These crystals were obtained using a screen condition that did not yield crystals in control drops [0.2 *M* ammonium sulfate, 30%(*w*/*v*) PEG 8000]. The presence of MIPs facilitated thaumatin crystal formation in a further 14 screen conditions where controls did not form, as detailed in Table 1 ▶. 14 of the 15 conditions did not contain tartrate, which is the most potent precipitant for thaumatin crystallization. Visualization of the modified MIPs is challenging. This is primarily owing to their altered consistency with respect to the original MIPs and also owing to the minute volume being added. Furthermore, if any precipitate forms within the crystallization drops (as in this instance) visualization is more improbable. The scale bar corresponds to 50 µm.

Having determined that the modified MIPs would function as nucleating agents and having determined the optimal conditions for their use, automated screening trials were commenced. Table 1 ▶ clearly indicates the ability of MIPs to increase the number of crystal ‘hits’ obtained. 12 new ‘hits’ were obtained for lysozyme, 15 for thaumatin (Fig. 2 ▶), four for trypsin and two for haemoglobin. The striking result is that haemoglobin-MIP, the MIP with the largest cavities, gave the most hits overall. For example, when screening with thaumatin at 30 mg ml^−1^, nine new conditions were discovered using haemoglobin-MIP, four with lysozyme-MIP and five with trypsin-MIP. The caveat in this instance being that the crystal ‘hits’ obtained were not as visually promising as those with lysozyme-MIP and trypsin-MIP. It is also interesting to note that the ‘hits’ obtained with haemoglobin-MIP often varied with respect to those obtained with lysozyme-MIP and trypsin-MIP.

Haemoglobin only yielded new hits with its own cognate MIP. This is not surprising as it has a greater molecular weight than lysozyme and trypsin. As such, the cavities formed on lysozyme-imprinted and trypsin-imprinted MIPs would have been far too small for the haemoglobin protein molecules. Conversely, haemoglobin-imprinted MIPs possess cavities that are larger than lysozyme, thaumatin and trypsin protein molecules, but do not prevent them from being entrapped within their cavities (in this instance it is possible that multiple protein molecules become entrapped within the large cavity). It appears that screening with an MIP containing larger cavities is beneficial.

The fact that lysozyme and trypsin are of similar molecular weight explains why their respective MIPs gave almost identical results. The lysozyme, trypsin and haemoglobin results illustrate this clearly. In the case of trypsin, the four ‘hits’ obtained were identical for each MIP tested. Screening trials with thaumatin were the exception. Although thaumatin was the only protein tested without its own cognate MIP, its similarity in molecular weight to trypsin and lysozyme would lead one to expect overlap in the ‘hits’ observed when screened with lysozyme-MIP and trypsin-MIP. However, very little correlation was observed. This result is indicative of factors other than cavity size (such as the cavity shape, charge *etc.*) having an influence on the induction of nucleation. The crystallization cocktail itself can impart a charge on specific parts of a protein, influencing its ability to form crystal contacts.

### 3.3. Automated optimization trials   

**Figure 3:**
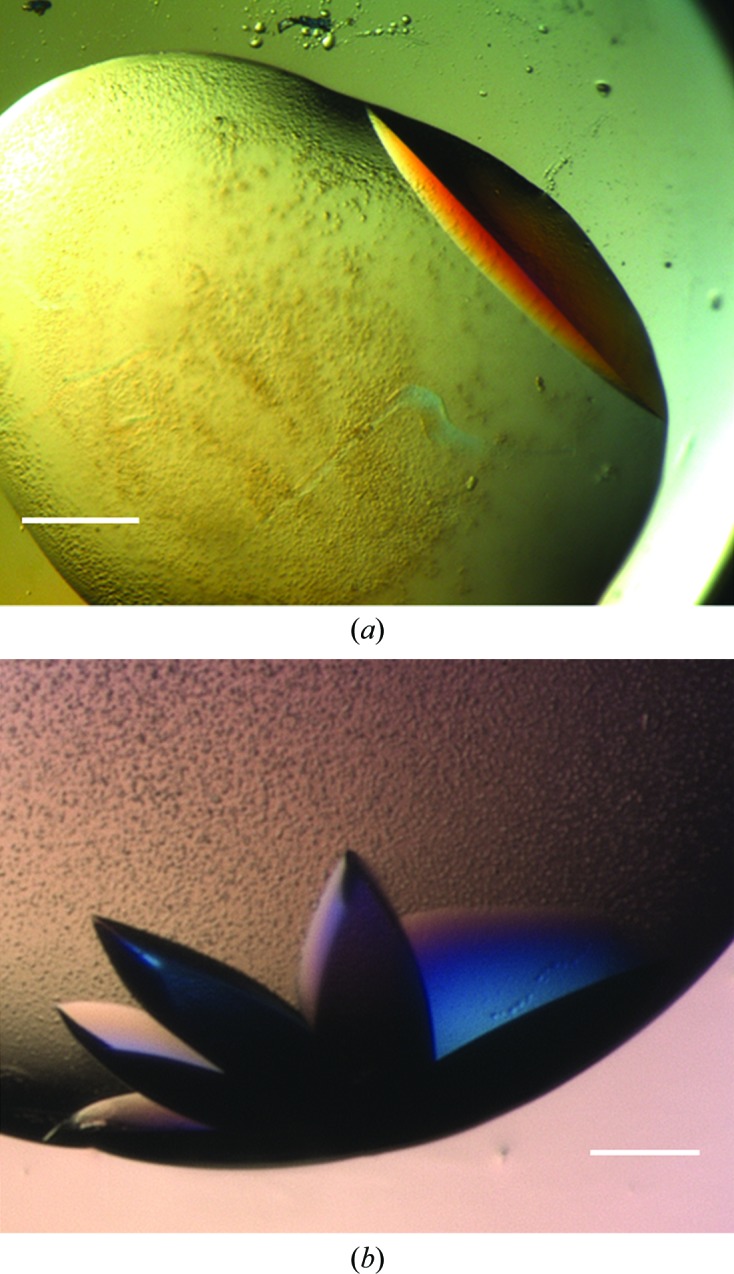
Pgp3 optimization. (*a*) A large, single Pgp3 crystal grown under metastable conditions using a cognate MIP. Single Pgp3 crystals were obtained using Pgp3-MIPs at dilutions of the original screen ‘hit’ between 50 and 70%. Corresponding controls remained clear. The scale bar corresponds to 75 µm. (*b*) Clusters of multiple Pgp3 crystals were reproducibly obtained using Pgp3-MIPs when the dilution of the original screen ‘hit’ was greater than 70%. The scale bar corresponds to 100 µm.

**Figure 4:**
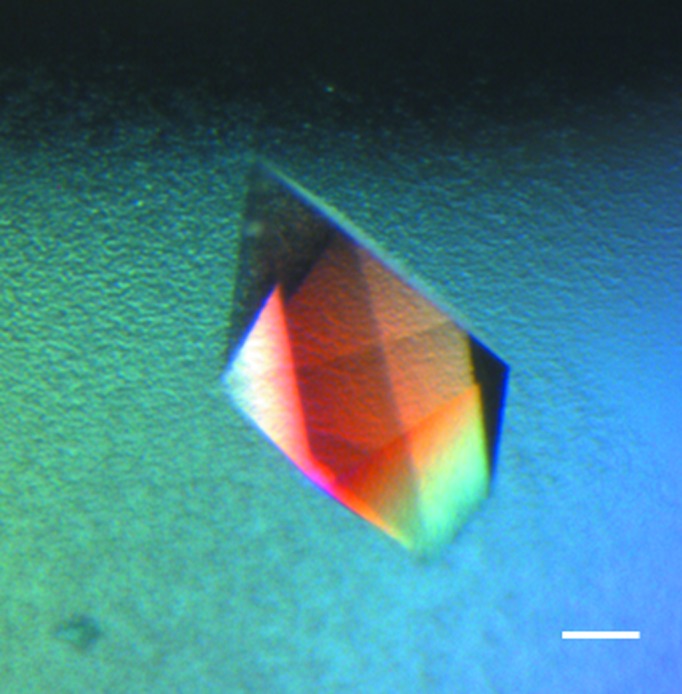
MIF optimization. A human macrophage migration inhibitory factor (MIF) crystal grown using a cognate MIF-MIP. This crystal was grown under metastable conditions (80% dilution of the original ‘hit’ condition) which would not normally yield crystals. The scale bar corresponds to 50 µm.

Table 2 ▶ illustrates the automated optimization results obtained. Both approaches for determining metastability were demonstrated to be suitable and the insertion of MIPs into crystallization drops at lower supersaturation reproducibly prompted the nucleation of trypsin, Pgp3 (Fig. 3 ▶) and MIF crystals (Fig. 4 ▶). Theoretically, the deeper into the metastable zone (*i.e.* the lower the extent of supersaturation) that nuclei find themselves, the slower their subsequent growth, resulting in improved crystal quality. The X-ray diffraction results obtained support this, with the diffraction resolution limits of Pgp3 and MIF crystals grown using cognate MIPs at lower supersaturation being repeatedly as good as, if not better than, crystals grown using MIPs at higher supersaturation and crystals grown without MIPs. In the case of Pgp3, the MIP-grown crystals diffracted to limits between 2.4 and 3.0 Å (whilst the control crystals attained 2.8–3.2 Å resolution). For MIF, 1.2 Å resolution was obtained compared with control crystals which diffracted to ∼1.4 Å resolution. No change in space group or unit-cell parameters was observed.

It was also determined that cognate MIPs were preferable for optimization trials. When haemoglobin-MIP was utilized to nucleate trypsin crystals it was not as potent as trypsin-MIP at lower supersaturation. As such, cognate MIPs, or MIPs created for a reference protein at as close a molecular weight as possible to the protein to be crystallized, are recommended for optimization trials.

**Figure 5:**
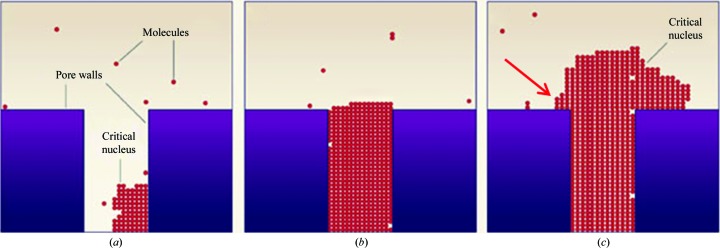
Nucleation in pores/cavities. Schematic illustration (modified from Frenkel, 2006 ▶) indicating the potential secondary nucleation sites formed when a protein crystal nucleates within a pore or cavity on a generic porous nucleating substrate. According to the theoretical model proposed by Page & Sear (2006 ▶), (*a*) the initial critical nucleus forms at the corner of the pore, (*b*) the pore is filled followed by subsequent growth out of the pore and (*c*) a second critical nucleus forms at the point where the protein aggregate growing out of the pore forms a junction with the nucleant surface. This model is based upon computer simulations, with the white voids observed being a consequence of the simulation process. A critical nucleus can comprise between ten and 100 protein molecules. It is possible that another secondary nucleation site can form at the location indicated by the red arrow. At higher levels of metastability there is sufficient protein to feed both nucleation sites. Furthermore, it is also possible that the protein aggregate growing from the pore may form a crystal itself.

As expected, when introduced into drops which would form crystals in any case, the MIPs only served to increase the rate and extent of nucleation, resulting in showers of microcrystals. As such, it is important to exploit the metastable region at lower supersaturation when optimizing. For example, at the metastable cusp (the concentration corresponding to conditions at and immediately below the supersolubility curve) the Pgp3 crystals obtained were clustered together (Fig. 3 ▶ *a*). It was only at lower supersaturation that the crystals obtained were single and larger in size (Fig. 3 ▶ *b*). A potential explanation for this phenomenon could be the creation of secondary nucleation sites in close proximity to the cavity which traps the protein molecules. Studies using atomic force microscopy have shown that MIP cavities can contain protein aggregates (El-Sharif *et al.*, 2014 ▶). The theoretical model for nucleation within pores as proposed by Page & Sear (2006 ▶) predicts a two-stage process in which a pore (or cavity) is initially filled with protein molecules starting at its corners, followed by growth out of the pore within the bulk solution. When the protein aggregate that forms within this cavity grows out of it, two secondary nucleation sites are created at the point where this aggregate meets the nucleant surface, all within a distance of a few nanometres (Fig. 5 ▶). At higher supersaturation there is more protein in solution to feed multiple nucleation sites, whilst at lower supersaturation growth at only one site is favourable. This phenomenon may be exacerbated by nucleants which have a high surface roughness.

### 3.4. Commercialization   

Having patented the design and application of MIPs for crystallization, and validated the modified MIPs for high-throughput trials, the way is now paved for commercialization. This will involve the production of a library of reference MIPs of varying molecular weights that could be used for the high-throughput screening and optimization of any protein, which is the ultimate goal of this research. As an optimal means for their application has now been determined, the resulting commercial product will be very simple to use and can be dispensed as an additive indiscriminately into trials (with molecular weight being the sole consideration when selecting an appropriately imprinted MIP). The MIPs are stored at 4°C and have a long shelf life. At most, they require vortexing if unused for more than a few weeks. Furthermore, their automated dispensing has been demonstrated with two robots. The Mosquito in particular has a fine-bore delivery system and as such we do not envisage tip blockage being an issue with other popularly used models.

## 4. Related literature   

The following reference is cited in the Supporting Information for this article: Asherie (2004 ▶).

## Supplementary Material

Supplementary materials and methods. . DOI: 10.1107/S1399004714027643/bw5424sup1.pdf

## Figures and Tables

**Table 1:** Crystal ‘hits’ when screening with MIPs Three MIPs were utilized for the screening of four model proteins. Details of all crystal ‘hits’ obtained in the presence of MIPs using Crystal Screen HT whilst the corresponding control drops remained clear are tabulated. The presence and absence of crystals is indicated by ‘Yes’ and ‘No’, respectively.

	MIP sample		
Screen condition	Haemoglobin-MIP	Lysozyme-MIP	Trypsin-MIP
Thaumatin at 30mgml^1^			
9	No	No	Yes
16	Yes	No	No
17	Yes	No	No
22	Yes	No	Yes
28	No	Yes	Yes
30	Yes	Yes	No
31	Yes	No	Yes
32	No	Yes	Yes
33	No	Yes	Yes
38	No	No	Yes
62	Yes	No	Yes
75	No	Yes	Yes
76	No	Yes	Yes
80	Yes	Yes	No
89	Yes	No	No
Lysozyme at 30mgml^1^			
4	Yes	Yes	Yes
14	No	Yes	No
16	Yes	No	No
18	No	No	Yes
43	Yes	No	No
45	Yes	No	No
46	No	Yes	Yes
60	No	Yes	Yes
73	Yes	Yes	No
75	Yes	Yes	Yes
78	No	Yes	Yes
94	No	No	Yes
Trypsin at 50mgml^1^			
14	Yes	Yes	Yes
42	Yes	Yes	Yes
43	Yes	Yes	Yes
46	Yes	Yes	Yes
Haemoglobin at 50mgml^1^			
45	Yes	No	No
82	Yes	No	No

**Table 2:** Trypsin automated optimization trials Table detailing the ability of trypsin-MIPs and haemoglobin-MIPs to induce the nucleation of trypsin crystals under metastable conditions. Each trial was repeated eight times, with the number indicating the number of repeats where the nucleating agent facilitated crystal formation whilst the corresponding controls remained clear. At higher supersaturation both MIPs suffice, whilst at lower supersaturation (deeper in the metastable zone) the cognate MIP was more potent and reliable.

	PEG concentration					
MIP sample	10%	11%	12%	13%	14%	15%
Trypsin-MIP	0	2	6	8	8	8
Haemoglobin-MIP	0	0	1	4	7	8
