# Supplementary material for: Automating the application of smart materials for protein crystallization
Source: Acta Crystallogr D Biol Crystallogr. 2015 Feb 26;71(Pt 3):534–40. doi: 10.1107/S1399004714027643 (PMC4356364; doi:10.1107/S1399004714027643)
Supplement: Supplementary file 1 [file d-71-00534-sup1.pdf]

BIOLOGICAL CRYSTALLOGRAPHY

Volume 71 (2015)

Supporting information for article:

Automating the application of smart materials for protein crystallization

Sahir Khurshid, Lata Govada, Hazim F. El-Sharif, Subrayal M. Reddy and Naomi E. Chayen

S1. MIP Conditioning

The individual MIPs were granulated using a 75 µm sieve (Inoxia Ltd., UK*).* 100 mg of the resulting MIPs were transferred into separate 1.5 ml eppendorf tubes and conditioned by washing with five 0.4 ml volumes of MilliQ water, followed by five 0.2 ml volumes of 10% (w/v):10% (v/v) SDS:Acetic acid (pH 2.8) and another five 0.2 ml volume washes of MilliQ water to remove any residual 10% (w/v):10% (v/v) SDS:Acetic acid eluent. Each wash was followed by a 3 minute centrifugation at 6000 rpm (RCF: 2419 x g) using an Eppendorf Mini-Spin Plus centrifuge. All supernatants were collected for spectrophotometric analysis to verify the extent of template removal and to ensure that the final water wash and SDS:Acetic acid eluent fractions did not contain any protein. This is important as it confirms that there would be no template protein within the MIPs that could leach out during later studies. The conditioned MIPs were then diluted in MilliQ water at ratios of 1:2, 1:3 and 1:5 (w/v) and stored at 4º C for crystallisation trials.

S2. MIP Characterisation

The subsequent rebinding effect of the conditioned and equilibrated MIPs and NIPs were characterized using a UV mini-1240 CE spectrophotometer (Shimadzu Europa, UK). The MIPs were treated with 0.3 mg/ml template protein solutions prepared in MilliQ water (using twice the volume of that of gels), mixed on a rotary vortex mixer for 15 seconds, allowed to associate at room temperature (22˚ C) for 20 minutes and then centrifuged at 6000 rpm (RCF: 2419 x g) for 3 minutes before the supernatants were collected. The MIPs were then washed four times with MilliQ water (again using twice the volume of that of gels). Each reload and wash step for the MIPs was followed by centrifugation at 6000 rpm (RCF: 2419 x g) for 3 minutes. All supernatants were collected for spectrophotometric analysis (at 404 nm for haemoglobin, 280 nm for trypsin, lysozyme, Pgp3 and MIF).

Any protein unaccounted for at this stage was deemed to be selectively bound to the MIP (or NIP) and determined by subtracting the protein levels observed in the supernatants from the initial load. Therefore, the molecular imprinting effect was characterised by calculating the rebinding capacity (Q) of protein to the polymer (mg/g) exhibited by the protein-specific MIP and the control NIP using the equation $Q = [C_i - C_r]\ V/g$, where C_i is the initial protein concentration, C_r is the recovered protein concentration (mg/ml), V is the volume of the initial solution (ml) and g is the mass of the polymers (g).

S3. 'Hit' Conditions for Automated Optimisation Trials

Pgp3 was crystallised at 7 mg/ml using 10% (wt/vol) PEG 2000 MME, 0.2 M Potassium Bromide, 0.2 M Potassium Thiocyanate, 0.1 M Sodium Cacodylate pH 6.5 and 3% (wt/vol) poly-ϒ-glutamic acid (low molecular weight ~400 kDa). MIF was crystallised at 11 mg/ml using 1.6 M ammonium sulphate, 3% (v/v), isopropanol and 0.1 M Tris pH 7.5. Trypsin was crystallised at 50 mg/ml using 16% (w/v) PEG 8000 and 0.1 M Tris pH 7.5.

S4. Determining Metastability

The metastable zone of the phase diagram lends itself to optimal crystal growth by providing existing crystal nuclei with a supersaturated environment which is ideal for their growth into crystals (Asherie, 2004). Metastability was determined robotically using two simple approaches. The first is conventional and involves dispensing a grid screen around a known crystallisation 'hit' condition, where the primary precipitant concentration is decreased in steps. For example, 50 mg/ml trypsin typically crystallises using 16% (w/v) PEG 8000 and 0.1 M Tris pH 8.0. By decreasing the PEG 8000 concentration in 1% steps one can determine conditions which correspond to metastable conditions (typically those immediately below where spontaneous crystallisation takes place). The Oryx robot was particularly adept in this case.

The second approach involved dispensing dilutions of the original 'hit' condition. For example, 7 mg/ml Pgp3 forms crystals in (a) 10% (wt/vol) PEG 2000 MME, 0.2M Potassium Bromide, 0.2M Potassium Thiocyanate, 0.1M Sodium Cacodylate pH 6.5 and 3% (wt/vol) poly-ϒ-glutamic acid (low molecular weight ~400kDa). By preparing dilutions of this 'hit' using deionised water (typically 5% steps, giving 95%, 90%, 85% dilutions and so on) it is possible to explore lower supersaturation and hence metastability.

Asherie, N. (2004). *Methods* **34**, 266-272.
